# Contribution of mTOR and PTEN to Radioresistance in Sporadic and NF2-Associated Vestibular Schwannomas: A Microarray and Pathway Analysis

**DOI:** 10.3390/cancers12010177

**Published:** 2020-01-10

**Authors:** Isabel Gugel, Florian H. Ebner, Florian Grimm, Stefan Czemmel, Frank Paulsen, Christian Hagel, Marcos Tatagiba, Sven Nahnsen, Ghazaleh Tabatabai

**Affiliations:** 1 Center for Neuro-Oncol., Comprehensive Cancer Center Tübingen Stuttgart, 72076 Tübingen, Germany; 2 Department of Neurosurgery, University Hospital Tübingen, 72076 Tübingen, Germany; 3 Centre of Neurofibromatosis and Rare Diseases, University Hospital Tübingen, 72076 Tübingen, Germany; 4 Interdisciplinary Division of Neuro-Oncol., University Hospital Tübingen, 72076 Tübingen, Germany; 5 Hertie Institute for Clinical Brain Research, Eberhard Karls University Tübingen, 72076 Tübingen, Germany; 6 Department of Neurosurgery, Alfried Krupp Hospital, 45131 Essen, Germany; 7 Quantitative Biology Center (QBiC), University of Tübingen, 72076 Tübingen, Germany; 8 Department of Radiation Oncology, University Hospital Tübingen, 72076 Tübingen, Germany; 9 Institute of Neuropathology, University Medical Center Hamburg-Eppendorf, 20251 Hamburg, Germany

**Keywords:** neurofibromatosis type 2, microarray analysis, radioresistance, signaling, vestibular schwannoma

## Abstract

The use of radiation treatment has increased for both sporadic and neurofibromatosis type 2 (NF2)-associated vestibular schwannoma (VS). However, there are a subset of radioresistant tumors and systemic treatments that are seldom used in these patients. We investigated molecular alterations after radiation in three NF2-associated and five sporadically operated recurrent VS after primary irradiation. We compared these findings with 49 non-irradiated (36 sporadic and 13 NF2-associated) VS through gene-expression profiling and pathway analysis. Furthermore, we stained the key molecules of the distinct pathway by immunohistochemistry. A total of 195 differentially expressed genes in sporadic and NF2-related comparisons showed significant differences based on the criteria of *p* value < 0.05 and a two-fold change. These genes were involved in pathways that are known to be altered upon irradiation (e.g., mammalian target of rapamycin (mTOR), phosphatase and tensin homolog (PTEN) and vascular endothelial growth factor (VEGF) signaling). We observed a combined downregulation of PTEN signaling and an upregulation of mTOR signaling in progressive NF2-associated VS after irradiation. Immunostainings with mTOR and PTEN antibodies confirmed the respective molecular alterations. Taken together, mTOR inhibition might be a promising therapeutic strategy in NF2-associated VS progress after irradiation.

## 1. Introduction

Vestibular schwannomas (VS) are the most common lesions of the cerebellopontine angle and occur with an incidence of 1:100,000 per year [1,2]. In rare cases, these tumors can appear bilaterally as typical hallmarks of the tumor prone disorder neurofibromatosis type 2 (NF2) [1,3]. Patients with NF2-associated VS not only get symptomatic earlier but also suffer additional benign nervous system tumors (e.g., meningiomas, non-VS schwannomas, and ependymomas) and/or are associated with other manifestations such as vascular disease [4,5]. Even though they are slow growing tumors, VS can cause hearing loss, tinnitus, dizziness, facial palsy, and, if large enough, hydrocephalus and death [1,3,4]. Established treatment options are microsurgery [6,7], radiation treatment [8,9], or, for patients with NF2-associated VS, systemic therapy (e.g., with bevacizumab) [10,11]. Particularly in young patients with NF2, good short-to-medium-term hearing results and growth control can be achieved by electrophysiological-guided microsurgery [7,12].

In contrast to sporadic VS, the use of radiosurgery in NF2-associated VS remains controversial due to a 20–40% chance of hearing impairment as a result of treatment [13,14] and the rare risk of malignant transformation [15]. Furthermore, radiosurgery has also been described to be less effective regarding tumor control rates in NF2-associated VS [14]. There is a 2–9% risk of tumor regrowth for sporadic tumors [16] and 12.5% for NF2-associated tumors [17]. However, this treatment modality is useful and an effective option for patients who are considered to be at high risk for surgical complications or in tumors that progress after surgery [18]. The molecular effects of irradiation on the biology of VS, particular in recurring or progressing tumors after radiation treatment, are not fully understood.

The role of the tumor suppressor gene *NF2*, which is located on chromosome 22q12 and its protein product moesin–ezrin–radixin-like protein (merlin) is well established in the biology of VS and NF2 [19,20,21]. A dysfunction of merlin (also known as schwannomin or neurofibromin 2) is a common mechanism in VS and other NF2-associated tumors [19,22,23,24,25,26]. In addition, merlin loss or inactivation leads to the dysregulation of signaling pathways that regulate cell survival, growth and proliferation [27,28,29,30,31].

However, the inactivation of the *NF2* gene is not found in every VS, which possibly indicates additional mechanisms that might be involved in tumorigenesis [3,32]. Gene expression profiling and pathway analysis in other studies have identified a high range of tumor-related candidate genes and pathways that contribute to the biology of VS [19,26,33,34,35,36,37,38,39]. None of these studies, however, have investigated differences between irradiated tumors and their controls. 

In the current study, we conducted a whole-genome microarray approach to identify statistically significant differences at the transcriptome level between recurrent irradiated VS and non-irradiated controls. We therefore used microdissected tumor samples of eight recurrent irradiated VS (five sporadic and three NF2-associated VS) and compared them with 49 non-irradiated controls (36 sporadic and 13 NF2-associated VS). In a canonical pathway analysis and immunohistochemical investigation, the aberrant activation of signaling pathways involved in tumor biology were observed.

## 2. Results

### 2.1. Clinical Data

Detailed clinical and summarized demographic data are given in Supplementary Table S1 and Table 1. Overall, the majority of patients had large (T3 and T4) tumors (96%). Only two tumors were small (T1 and T2) and associated with NF2. Pre-operatively, hearing was functional (Gardner and Robertson Scale (G–R) grades 1 and 2) in 28%, non-functional (G–R grades 3 and 4) in 16%, and 32% were deaf (G–R grade 5). Irradiated sporadic ears exhibited worse G–R grades compared to their non-irradiated controls. Facial nerve function was good/excellent (House and Brackmann Facial Nerve Grading System (H–B) grades I and II) in the vast majority, 84%, of patients.

Before surgery, most patients suffered from hearing loss (88%), followed by trigeminal nerve dysfunction (hyp-/dysesthesia, neuralgia, reduced/lost corneal reflex, 46%), gait disturbances (37%), tinnitus (35%), and facial palsy (34%), and (25%) had an event of sudden hearing loss.

We observed no significant difference in sex, the age at time of diagnosis/surgery, tumor size, the presence of tinnitus, gait disturbances and vertigo, the dysfunction of the trigeminal nerve, or the event of sudden hearing loss between the groups. Nevertheless, of the predictor variables, only facial nerve function (H–B grade) was significantly (*p* = 0.02) worse in both irradiated sporadic and NF2-associated (mean H–B grade 3 ± 1.4) compared to the non-irradiated sporadic and NF2-associated group (mean H–B grade 1.4 ± 0.9).

All patients with NF2 exhibited mutations in the *NF2* gene (Supplementary Table S1).

Radiation treatment was carried out in various fractionations, but mainly radiosurgically.

### 2.2. The mTOR and PTEN Signaling Seem to Be of Potential Interest in Recurrent Irradiated NF2-Associated Vestibular Schwannomas

A microarray analysis was performed, as described in detail in the Material and Methods Section 4.3 and Section 4.4. By using the statistically significant criteria of a minimum of two-fold log fold change and a multiple adjusted *p*-value < 0.05, we identified 195 shared, differentially expressed genes in the irradiated vs. the non-irradiated NF2- and sporadic-related comparisons. A total of 128 of these genes were significantly upregulated, whereas 67 genes were downregulated. Detailed results for the differential expression (DE) analysis across all comparisons are given in Supplementary Table S2.

Due to the small number of cases, the comparison between previously irradiated recurrent or progressive NF2-associated and sporadic VS (“NF2_irradiated vs. Sporadic-irradiated”) was not further considered for the analysis.

To identify canonical pathways that may be altered in irradiated VS, we performed an ingenuity pathway analysis (IPA, Ingenuity^®^ Systems, www.ingenuity.com) by using these differentially expressed genes (all regulated pathways are shown in detail in Supplementary Table S3). We focused on pathways that may influence radioresistance through the regulation of cell cycles, apoptosis, angiogenesis and proliferation and with a known influence of merlin or a relation to vestibular schwannoma [43,44]. Included pathways and their regulations are listed in Table 2.

Angiogenesis and proliferation signaling such as vascular endothelial growth factor (VEGF), neuregulin (NRG) and human epidermal growth factor receptor-2 and 3 (ErbB2–ErbB3) signaling was downregulated in both sporadic and NF2-associated VS after irradiation. The radiation-induced apoptosis signaling represented by stress-activated protein kinase/c-Jun NH2-terminal kinase (SAPK/JNK) and tumor protein p53 (p53) was upregulated in irradiated sporadic and downregulated in irradiated NF2-associated tumors. Its mediator Rac signaling was only upregulated in NF2-associated VS after irradiation, whereas the mitogen-activated protein kinase (MAPK/ERK) pathway was downregulated and the downstream target p38 MAPK was not regulated. The cell cycle G1/S checkpoint was downregulated in irradiated NF2-associated VS but not in sporadic VS. The deregulation of cell cycle signaling (e.g., ataxia-telangiectasia mutated kinase (ATM), cell cycle checkpoint G2/M) was not detected.

We assumed the mammalian target of rapamycin (mTOR) and phosphatase and tensin homolog (PTEN) signaling as core signaling due to their known relevance in the tumorigenesis of VS and radioresistance. Progressive or recurrent NF2-associated VS showed a significant downregulation of PTEN signaling and an upregulation of mTOR signaling that were different from sporadic tumors after irradiation. For the key genes *mTOR* and *PTEN*, microarray results showed a significant downregulation of *PTEN* in both recurrent NF2-associated (one out of four microarray probe sets for PTEN) and sporadic VS (four out of four microarray probe sets for PTEN) after irradiation. The expression of *mTOR* was not significantly different in any group in irradiated tumors (Supplementary Figure S1).

**Figure 1:**
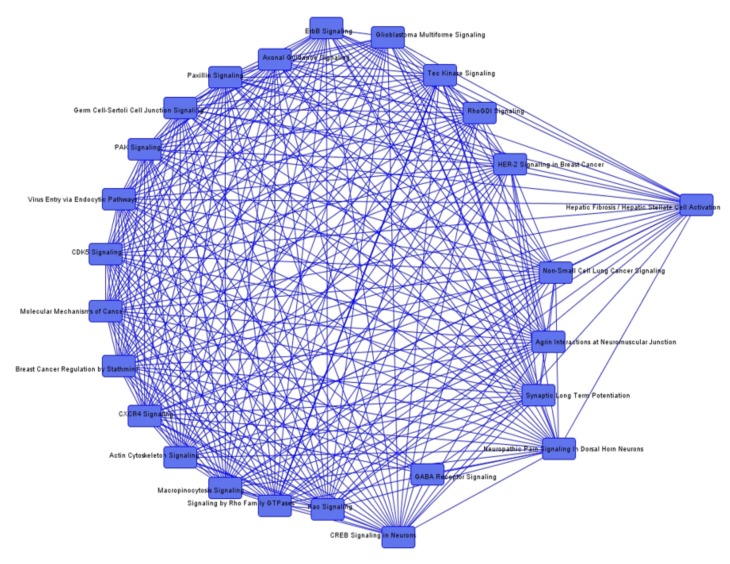
Overlapping canonical pathways in irradiated recurrent vs. non-irradiated neurofibromatosis type 2 (NF2) associated vestibular schwannomas (VS).

**Figure 2:**
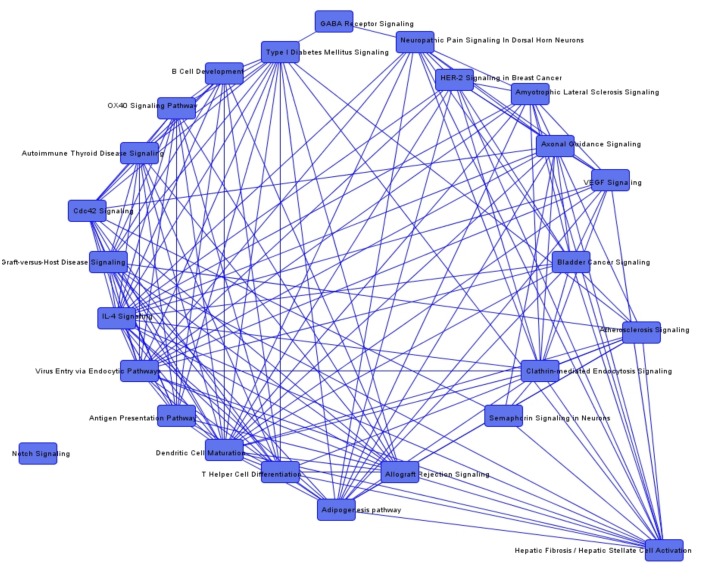
Overlapping canonical pathways in irradiated recurrent vs. non-irradiated sporadic vestibular schwannomas (VS).

**Figure 3:**
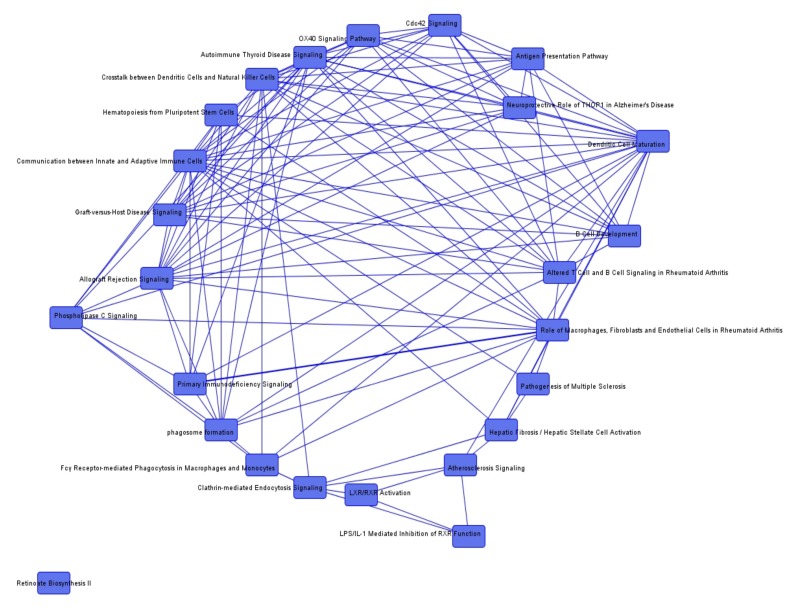
Overlapping canonical pathways in non-irradiated sporadic vs. neurofibromatosis type 2 (NF2) associated vestibular schwannomas (VS).

To identify common genes among the pathways, we additionally determined overlapping canonical pathways. We could not detect any overlapping genes between the pathways that were conclusive in the investigated context of irradiation. Nevertheless, overlapping canonical pathways in the group comparisons between irradiated and non-irradiated tumors are demonstrated in Figure 1, Figure 2 and Figure 3.

### 2.3. Irradiated NF2-Associated VS Revealed Significant Lower PTEN Expression Intensity Compared to Non-Irradiated or Sporadic Tumors

**Figure 4:**
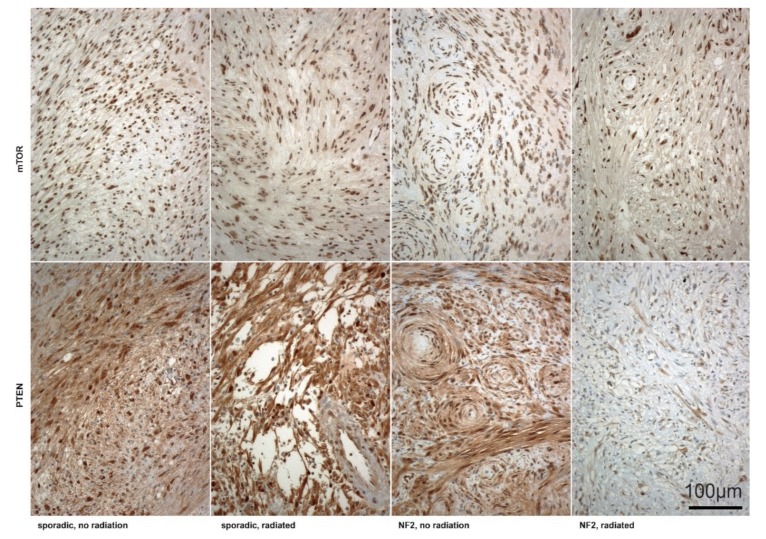
Immunostaining for mammalian target of rapamycin (mTOR) (upper row) and phosphatase and tensin homolog (PTEN) (lower row) in non-irradiated compared to irradiated VS in the two comparison groups (NF2 vs. sporadic). Scale bar = 100 µm.

An immunohistochemical analysis was performed in seven irradiated samples (three NF2 and four sporadic) and in all (49) controls (36 sporadic and 13 NF2-associated VS). All samples were positive for S100 staining. Apart from two sporadic non-irradiated tumors with weak (20% of cells) or absent (0% of cells) immunoreactivity for mTOR, all other samples (irradiated and non-irradiated) were highly (100%) stained positive. PTEN expression intensity (0–3) was significantly (*p* = 0.042) lower in irradiated NF2-associated VS (median of 2) compared to non-irradiated tumors with NF2 (median of 3). The signal intensity in both sporadic non-irradiated (median of 3) and irradiated samples (median of 3) showed no significant difference (*p* > 0.05). Examples of the comparison groups are illustrated in Figure 4. Individual immunohistochemical results are outlined in supplementary Table S1 and are demonstrated in scatterplots in Supplementary Figures S2 and S3.

## 3. Discussion

Previously, several studies have investigated the transcriptome in different types of VS (Table 3) [26,33,34,39,45,46,47,48], but none of them have focused on the results of irradiated tumors versus their controls. Especially, the molecular behavior of recurrent or progressive sporadic and NF2-associated VS after irradiation has not been examined in detail. The development of radiosensitizers to avoid continued growth would be desirable.

We therefore aimed to investigate the molecular mechanisms of acquired resistance to radiation therapy in a single center cohort. In our study, we performed a microarray analysis on five sporadic and three NF2-associated VS that were recurrent or progressive following radiotherapy, and we compared them, amongst other comparisons, to 49 (36 sporadic and 13 NF2-associated) non-irradiated tumors. In the ingenuity pathway Analysis, we focused on pathways that have been previously described in irradiated brain tumors and VS [43,44], and we corroborated our main findings with immunohistochemistry.

We observed complex deregulation of these signaling pathways and identified the PTEN/mTOR signaling deregulation in merlin-inactive, NF2-associated VS as a particular finding in these recurrent/regrowing tumors after irradiation. The previous studies in this field are outlined in Table 3.

### 3.1. Low Proliferation Signaling Reveals Radioresistance

The neuregulin (NRG-1)/ErbB2–ErbB3 signaling and its genes have been well described and play an important role in the tumorigenesis of VS.

The neuregulin-1(NRG1) protein itself is important for Schwann cells and their precursors in the process of cell survival, differentiation, and proliferation during their development. High levels of NRG1 and its isoforms favor the development of human peripheral nerve sheath and VS cell proliferation [49,50,51,52], and NRG1 has been found to upregulated in a microarray analysis of VS [48].

The binding of NRG1 to its receptors ErbB2 and ErbB3 leads to the activation of intracellular signaling, including the PI3K/AKT and mTOR pathway, influencing Schwann cell (SC) proliferation, migration, differentiation and survival [53]. Thus, inhibition with antibodies that are directed against neuregulin-1 (anti-NRG1) or its receptors (trastuzumab) reduces SC proliferation in primary VS cell cultures [54]. Despite the radiation-resistant properties of VS cells, Hansen et al. found that sensitivity of them to irradiation depends on their proliferation capacity, and this can be influenced by the activity of NRG1/ErbB2 signaling [54].

Additionally, the induction of NRG-1 increases radiosensitivity by promoting ErbB2 signaling and enhancing the proliferation of cells [54].

Low proliferation potential in recurrent VS after irradiation was detected by Lee et al., demonstrating lower levels of proliferating cell nuclear antigen (PCNA) compared to controls [55].

In our data, neuregulin-1 and ErbB2–ErbB3 signaling was significantly downregulated in both sporadic and hereditarily-irradiated VS. Under consideration of the described literature, these irradiated tumors possibly exhibited a low proliferative tendency and were thereby less sensitive to irradiation. This finding needs to be verified by further analysis. A possible treatment with artificial neuregulin could conceivably enhance proliferation over ErbB2 signaling.

### 3.2. Suppression of Angiogenesis Mediators as an Effect of Irradiation

Vascular endothelial growth factor (VEGF or VEGF-A) is expressed in nearly all VS [56,57,58]. The inhibition of VEGF with bevacizumab, a monoclonal antibody, is an established treatment modality for NF2-associated VS and induces the regression of tumor volume and even improves hearing [56,59].

The use of bevacizumab combined with radiation therapy (RT) has been found to improve tumor control rate and to allow for a reduction of radiation dose and radiation-related toxicity in a NF2 schwannoma model; radiosensitivity was presumably enhanced due to normalization of tumor vasculature and improved perfusion and oxygen delivery [60].

Our data show that VEGF signaling was downregulated in both sporadic and NF2-associated VS, probably as an effect of irradiation rather than medication, because none of our NF2 patients received systemic therapy with bevacizumab before surgery or irradiation.

### 3.3. Cell Cycle Mediation and Regulation of Apoptosis

Irradiation is known to cause DNA damage through different mechanisms. Cell cycle arrest and apoptosis are predominantly regulated by the p53 transcription factor. DNA damage that is induced by radiation activates p53, which in turn activates the proapoptotic signaling. Intact or induced p53 was found to increase radiosensitivity in a glioblastoma cell model [61]. Multiple studies have shown that p53 is unlikely to contribute to the pathogenesis of VS [62,63]. In our cohort, both sporadic and NF2-associated non-irradiated tumors exhibited no regulation of p53, but irradiated tumors did. p53 was downregulated in NF2-associated VS, but it was upregulated in sporadic VS after irradiation indicating that p53 plays more of a role for NF2-associated than for sporadic tumors in radioresistance.

Radiation-induced apoptosis and/or cell cycle arrest can also be regulated by the SAPK/JNK pathway, a downstream signaling mediated by Rac [64], which, in turn, is negatively regulated by merlin [65]. Active merlin normally inhibits Rac-dependent signaling [66]. According to our data, radiation-induced apoptosis via the stress-activated protein kinase/c-Jun NH2-terminal kinase (SAPK/JNK) pathway was upregulated in sporadic and downregulated in NF2-associated VS after irradiation, which may indicate that apoptosis is reactively activated in sporadic and inhibited in NF2-associated VS. However, the mechanisms remain unclear. The regulation of the DNA cycle and its repair mechanism after irradiation, e.g., the phosphatidyl-inositol kinase-related protein ATM, was not or unspecifically altered.

### 3.4. PTEN and mTOR Signaling—Core Pathway in Merlin-Inactive and Progressive NF2-Associated Tumors after Irradiation

Among the detected pathways, the PTEN and mTOR pathways, which are known to be effective targets in genetic tumor syndromes and cancer, are specifically deregulated in merlin-inactive NF2-associated VS after irradiation, with the PTEN pathway being down and mTOR pathway being upregulated. Therefore, inactivation or downregulation of PTEN, as seen in our microarray and pathway analysis results, leads to the activation of mTOR and the enhancement of specific mRNAs that are crucial for cell growth and proliferation [67]. In merlin-deficient schwannomas, mammalian target of rapamycin complex 1 (mTORC1) is constitutively activated, and mTOR inhibitors such as rapamycin (sirolimus) and its rapalogs (e.g., everolimus) influence vestibular schwannoma cell proliferation [28,68,69,70]. The pharmacological targeting of mTORC1 either results in the direct inhibition of tumor growth [28,71] or the indirect over reduction of angiogenesis via VEGF [72]. Nevertheless, a conducted prospective, open-label phase II study in ten NF2 patients with progressive VS treated with everolimus could not confirm this antitumor activity response by in vitro models [73].

The dual inhibition of the PI3K/AKT/mTOR pathway by inhibitors (BEZ235, PKI-578) has been shown to increase cell death and decreases cell viability in schwannoma cell line models (HEI-293) [39].

The *NF2* gene product merlin acts as a negative regulator of *mTORC1* [28]. As an upstream target of the tuberous sclerosis complex (TSC), merlin may function as the major mTORC1 regulator in the cell [28]. Congruent to TSC null cells, merlin-deficient cells with a loss of the tumor suppressor function end up in high levels of mTORC1 signaling and thereby in a decrease of proliferation rates [28].

Therefore, in vivo and in vitro, *mTORC1* is a critical regulator of schwannoma growth, and mTOR inhibition may open up new opportunities for targeted therapies to stop the growth in these tumors [28].

The tumor suppressor gene *phosphatase and tensin homolog (PTEN)* is known to be frequently mutated in human cancers and some tumor syndromes. The loss of *PTEN* by mutations promotes the hyperactivation of AKT, which in turn favors cellular growth, survival and proliferation through mTOR and thus increases radioresistance [74]. In contrast, the enhanced expression of PTEN or the transfer of the *PTEN* gene in glioma cells sensitized the cells to radiation [75,76]. Congruent to its tumor suppressor function and as a negative regulator of the PI3K/AKT/mTOR pathway, the downregulation/inactivation of PTEN results in an activation of this pathway and thereby supports cell growth and survival.

In a transgenic mice model, Keng et al. 2012 [77] revealed that both the inactivation of the *PTEN* and *NF1* genes in Schwann cells, and Schwann cell precursors enhance the development of neurofibromas and additionally support the progression from a low- grade to a high-grade tumor. Consequently, *PTEN*-regulated pathways are major tumor-suppressive barriers to neurofibroma progression and favored malignant transformation in these tumors [77,78,79].

On the other hand, in a study of Pattje et al. [80], PTEN was found to function as a mediator of radiosensitivity in an in vitro head and neck cancer model, and PTEN activation correlated with poor response to radiation treatment. Controversially, the colleagues found out that PTEN expression correlated with high levels of AKT signaling, despite normally being an inhibitor of AKT [80]. Therefore, this mechanism could explain the radioresistant effect of PTEN, and this may be tumor-specific.

As a tumor suppressor, *PTEN* also acts as a maintainer of genome stability and has a role in DNA repair. Irradiation-induced DNA damage signaling or failures in DNA repair have been seen in tumor cells with lower expression levels of PTEN and increased levels of AKT [81,82].

### 3.5. Limitations of the Current Study

Despite the high concordance of microarray results and validation methods such as RT-PCR, we were unfortunately unable to validate our findings by confirmative RT-PCR due to a lack of residual tumor tissue. This would be beneficial, as RT-PCR is a very accurate technology with a greater dynamic range compared to microarrays and is therefore often used to validate the trends that are obtained by array experiments. Consequently, our results require further verification by a separate analysis, preferably on a larger cohort using a state-of-the-art transcriptomics such as RNA-sequencing.

## 4. Material and Methods

### 4.1. Patients and Tumors

Vestibular schwannoma tumor samples were retrospectively collected from 67 patients (39 males and 28 females) during their first surgical resection at the University Hospital Tübingen. Seven controls of vestibular nerve tissue were obtained post mortem and used as controls. Among the tumor samples, 36 were sporadic, 13 were NF2-associated, 9 were cystic, and 9 were pre-irradiated regrowing VS comprising five sporadic, one was cystic, and three were NF2-associated tumors. Tumor recurrence time after irradiation was over 3 years and pseudoprogression was excluded by regular imaging.

All samples were collected at the Department of Neurosurgery, University Hospital Tübingen, between July 2007 and July 2010. The use of tissue and clinical data was approved by the ethics committee (No 236/2009BO2) of the Eberhard Karls University Tübingen, Germany.

Tumor samples were microdissected by the retrosigmoid approach and were immediately frozen and stored at −80 °C. A conventional neuropathological examination with paraffin-embedded tissue sections histologically confirmed the diagnosis (as schwannoma WHO grade I). For all NF2-associated tumors, a mutation analysis was performed, as previously described [83].

Clinical data were analyzed by using the clinical records prior to surgery. Tumor size was graded according to the Hannover classification system [40]. Hearing data were assessed by pure tone average (PTA) and speech discrimination score (SDS), and hearing function was classified according to the Gardner Robertson (G–R) scale [41]. Facial motor function was determined by using the House and Brackmann (H–B) classification [42].

### 4.2. RNA Extraction and RNA Integrity Number (RIN)

Total RNA was extracted from non-irradiated and irradiated tumors by using a RNeasy**^®^** Microarray Tissue Mini Kit System (Qiagen, Valena, CA, USA) according to the manufacturer′s instructions and stored at −80 °C. Quantitative and qualitative RNA was verified by a lab-on-a-chip-system by using the Agilent 2100 Bioanalyzer (Agilent Technologies Inc., Palo Alto, CA, USA). The RNA quality and quantity were evaluated by using the RNA integrity number (RIN) that was calculated for all samples. The samples with a RIN higher or equal to 5 were selected for analysis.

### 4.3. cDNA Microarray Analysis

A microarray analysis was initially performed for all samples (74) to detect differentially expressed genes between groups of interest. For expression profiling, 100 ng of total RNA was isolated, linearly amplified, and biotinylated with the GeneChip**^®^** HT 3′IVT Express Kit (Affymetrix, Santa Clara, CA, USA) according to the manufacturer’s instructions. Fifteen micrograms of labeled and fragmented cRNA were then hybridized onto Human Genome U219 Gene Chip^®^ arrays (Affymetrix). Hybridization, washing, staining and scanning were automatically performed in a GeneTitan^TM^ instrument (Affymetrix). Scanned images were subjected to visual inspection to control for hybridization artifacts and proper grid alignment, and they were analyzed with AGCC 3.0 (Affymetrix) to generate CEL files.

### 4.4. Statistical Array Analysis

All subsequent DE analysis steps were performed in the language R (version 3.1.1) [84] mainly with the packages “affy” (version 1.42.3) [85] and “limma” (version 3.20.9) [86,87]. Initially, the expression data from all 74 chips were quantile normalized with RMA (robust multichip average) [88]. The RMA-normalized expression values for each probe set are, together with the raw Affymetrix CEL files, available in Gene Expression Omnibus (GEO) with the accession number GSE141801.

The signal values were then averaged for the individual subgroups, and differences in expression levels of genes between subgroups were extracted as contrasts. These steps were done with standard limma functions including an empirical Bayes step [89] and a correction step for multiple testing to determine significance for DE analysis based on a multiple adjusted *p* value < 0.05, detailing that the false discovery rate (FDR) was controlled at 5% by using the “Benjamini–Hochberg” (BH) method [90]. That means that if all genes with a multiple adjusted *p* value < 0.05 were selected as differentially expressed, then the expected proportion of false discoveries in the selected group was controlled to be less than 5%. Thirteen comparisons were extracted from the model as major contrasts. Statistics for these contrasts including log2 ratios and nominal and multiple adjusted *p* values for each probe set are given in Supplementary Table S2. Three of them, namely “NF-irradiated vs. NF2,” “Sporadic-irradiated vs. Sporadic,” and “NF2 vs. Sporadic” were subsequently used for IPA analyses.

In order to include genes for web tool analysis, those genes with at least a 2-fold positive or negative change of expression in the respective comparison and adjusted *p* < 0.05, fold changes were kept for IPA analysis; for all other genes, fold changes were set to a missing value (NA).

### 4.5. Pathway Analysis

For the identification of gene networks and pathways that may be deregulated in VS, we performed an ingenuity pathway analysis (IPA, Ingenuity**^®^** Systems). The dataset with differentially regulated genes and their corresponding expression values, as detailed above, was uploaded into the IPA application. The deregulated genes were overlaid onto a global molecular network developed from information in the Ingenuity Pathway Knowledge Base. The networks and pathways of the focus genes were then algorithmically generated based on their connectivity. All edges are supported by at least one reference from the literature, from a textbook, or from canonical information stored in the Ingenuity Pathways Knowledge Base. The software *p*-value cutoff was decreased from 0.05 to ≤0.0001. Abbreviations of the signaling and proteins name were based on gene symbol.

After a primary analysis of the data, we focused on the comparison between sporadic and NF2-associated recurrent irradiated VS compared to their non-irradiated controls. Thus, the total tumor number decreased from 74 to 57 for further immunohistochemical and clinical analysis.

### 4.6. Immunohistochemistry (IHC)

All samples were stained with S100 to confirm diagnosis of a schwannoma. To corroborate the key pathway results from the microarray analysis, we carried out immunohistochemistry on slides from seven irradiated samples (4 sporadic and 3 NF2-associated recurrent irradiated VS) and the 49 non-irradiated controls (36 sporadic and 13 NF2-associated VS). One irradiated sporadic sample was not available for the analysis. Immunohistochemistry was performed in an automated stainer (Ventana Medical Systems, Tucson, AZ, USA) by using a standard antigen retrieval protocol (cc1m). The antibodies (mTOR: antibodies-online # ABIN747158, PTEN (138G): cell signaling # 9559) were both diluted 1:100. Staining was scored based on intensity for PTEN (0–3) and the proportion of tumor cells (0–100%) for mTOR. Subsequently, the tumors were categorized into 3 groups: PTEN negative (0, +), PTEN positive (+, ++, ++ [+], +++), and mTOR positive >50%.

### 4.7. Statistical Clinical Data Analysis

A binomial logistic regression was performed to identify differences between irradiated and non-irradiated tumors regarding sex, age, tumor size (Hannover classification [40]), the presence of tinnitus, vertigo, gait disturbances, the dysfunction of trigeminal and facial nerves (H–B grade), and the event of sudden hearing loss.

## 5. Conclusions

The combined downregulation of PTEN signaling and the upregulation of mTOR signaling in the merlin-deficient VS of patients with NF2 after irradiation might indicate a promising therapeutic target for pharmacotherapy. Thus, mTOR-inhibitors could be considered in these patients who suffer from continued tumor growth after irradiation.

## Figures and Tables

**Table 1:** Clinical and demographic data of 49 non-irradiated and eight irradiated vestibular schwannomas (VS).

	Non-Irradiated VS (49)		Irradiated VS (8)	
	SPO (36)	NF2 (13)	SPO (5)	NF2 (3)
Sex				
male	23	6	1	3
female	13	7	4	0
Operation side				
left	24	5	3	0
right	12	8	2	3
Age at diagnosis in year	44 ± 12	25 ± 8	49 ± 10	32 ± 12
(mean ± std, range)	25–76	11–40	33–62	24–49
Age at surgery in year	45 ± 12	25 ± 7	51 ± 12	33 ± 11
(mean ± std, range)	25–76	11–40	33–67	25–49
Age at radiation treatment			49 ± 11	29 ± 12
(mean ± std, range)			31–64	20–47
Tumor size (Hannover classification system [40]) pre-op				
T1	0	1	0	0
T2	0	1	0	0
T3	11	3	1	1
T4	25	8	4	2
G–R Class [41] pre-op				
1	4	1	0	0
2	11	0	0	0
3	8	0	2	0
4	0	0	0	0
5	4	9	3	2
Not available	9	3	0	1
H–B Class [42] pre-op				
I	28	8	2	0
II	8	1	1	0
III	0	1	1	1
IV	0	1	0	0
V	0	2	1	2
Clinical symptoms pre-op				
Tinnitus	17	2	1	0
Event of sudden hearing loss	12	2	0	0 2
Vertigo	19	3	4	0
Gait disturbances	13	8	0	1
Trigeminal nerve dysfunction (hyp-/dysesthesia, reduced/loss of corneal reflex, neuralgia)	16	6	3	

G–R: Gardner and Robertson Scale [41], H–B: House and Brackmann Facial Nerve Grading System [42]; NF2: neurofibromatosis type 2 association; SPO: sporadic.

**Table 2:** Important deregulated signaling pathways after irradiation of sporadic and neurofibromatosis type 2 (NF2)-associated tumors versus controls.

Ingenuity Canonical Pathways	Regulation		
	SPO_RAD-SPO	NF2_RAD-NF2	SPO-NF2
Notch signaling	1	nr	nr
TGF-β signaling	1	nr	nr
Wnt/β-catenin signaling	0	−1	nr
PTEN signaling	0	−1	nr
IGF-1 signaling	−1	−1	nr
VEGF signaling	−1	−1	nr
cMet/HGF signaling	−1	−1	nr
ErbB2–ErbB3 signaling	−1	−1	nr
Neuregulin signaling	−1	−1	nr
EGF/EGFR signaling	1	−1	nr
PDGF signaling	1	−1	nr
p53 signaling	1	−1	nr
STAT3 Pathway	1	−1	1
NF-κB signaling	1	−1	−1
FGF signaling	1	−1	−1
mTOR signaling	0	1	−1

NF2_RAD-NF2: NF2-associated irradiated VS versus (non-irradiated) NF2-associated VS; SPO-NF2: (non-irradiated) sporadic VS versus (non-irradiated) NF2-associated VS; SPO_RAD-SPO: sporadic irradiated VS versus (non-irradiated) sporadic VS; nr = not represented in this group; 0 = no regulation change, −1 = significantly downregulated; and +1 = significantly upregulated. TGF-ß = transforming growth factor beta; Wnt/ß-catenin = proto-oncogene protein Wnt-1/beta-catenin; PTEN = phosphatase and tensin homolog; IGF-1 = insulin-like growth factor 1; VEGF = vascular endothelial growth factor; cMET = tyrosine-protein kinase Met/HGF = hepatocyte growth factor receptor; ErbB2 = receptor tyrosine-protein kinase erbB-2, aka human epidermal growth factor receptor 2, HER2; ErbB3 = receptor tyrosine-protein kinase erbB-3, aka human epidermal growth factor receptor 3, HER3; EGF/EGFR = epidermal growth factor/epidermal growth factor receptor; PDGF = platelet-derived growth factor; p53 = tumor protein p53, aka cellular tumor antigen p53; STAT3 = signal transducer and activator of transcription 3; NF-κB = nuclear factor kappa-light-chain-enhancer of activated B-cells; FGF = fibroblast growth factor; mTOR = mammalian target of rapamycin, aka mechanistic target of rapamycin.

**Table 3:** cDNA Microarray analysis in the field of vestibular schwannoma research.

Methods	No of Tumor Samples	No and Type of Control Samples	Author, Year
cDNA Microarray, RT-PCR	31 CYS-VS 6 SPO-VS	No	[45]
cDNA Microarray, Cell cultures, Western Blotting	36 SPO-VS 13 NF2-VS	7 vestibular nerves	[39]
cDNA Microarray, RT-PCR, MLPA analysis of NF2 LOH analysis	28 SPO-VS 3 NF2-VS	2 auricular nerves 2 cervical nerves 1 facial nerve 1 vestibular nerve 1 nerve from the VIII cranial pair 1 commercial normal human adult Schwann cell (HSC) RNA	[48]
cDNA Microarray, RT-PCR, IHC	15 SPO-VS 7 CYS-VS 3 RAD-VS	3 tibial nerves	[33]
cDNA Microarray, RT-PCR, IHC	13 VS	No	[46]
cDNA Microarray	16 SPO-VS	3 vestibular nerves	[34]
cDNA Microarray, RT-PCR	11 SPO-VS	11 blood samples	[47]
cDNA Microarray, RT-PCR, IHC	3 SPO-VS 1 CYS-VS 3 RAD-VS	1 vestibular nerve	[26]
cDNA Microarray, IHC	36 SPO-VS 13 NF2-VS 9 CYS-VS 9 RAD-VS	7 vestibular nerves	Current study

cDNA: complementary DNA; CYS-VS: cystic vestibular schwannoma; IHC: immunohistochemistry; LOH: loss of heterozygosity; MLPA: multiplex ligation-dependent probe amplification; NF2-VS: neurofibromatosis type 2-associated vestibular schwannoma; RAD-VS: irradiated vestibular schwannoma; RT-PCR: real-time polymerase chain reaction; SPO-VS: sporadic vestibular schwannoma.

## Supplementary Materials

The following are available online at https://www.mdpi.com/2072-6694/12/1/177/s1, Figure S1: Boxplots for *mTOR* (A) and *PTEN* (B) expression, Figure S2: Scatterplots of immunohistochemical expression of mTOR, Figure S3: Scatterplots of immunohistochemical expression of PTEN, Table S1: Detailed clinical data of the 57 tumor samples, Table S2: Final table of the microarray analysis, Table S3: Final table of all deregulated canonical pathways (IPA analysis).
