# Supplementary material for: Contribution of mTOR and PTEN to Radioresistance in Sporadic and NF2-Associated Vestibular Schwannomas: A Microarray and Pathway Analysis
Source: Cancers (Basel). 2020 Jan 10;12(1):177. doi: 10.3390/cancers12010177 (PMC7016954; doi:10.3390/cancers12010177)
Supplement: Supplementary file 1 [file cancers-12-00177-s001.zip › cancers-686519-supplementary_corr.docx]

**Supplementary Materilas**


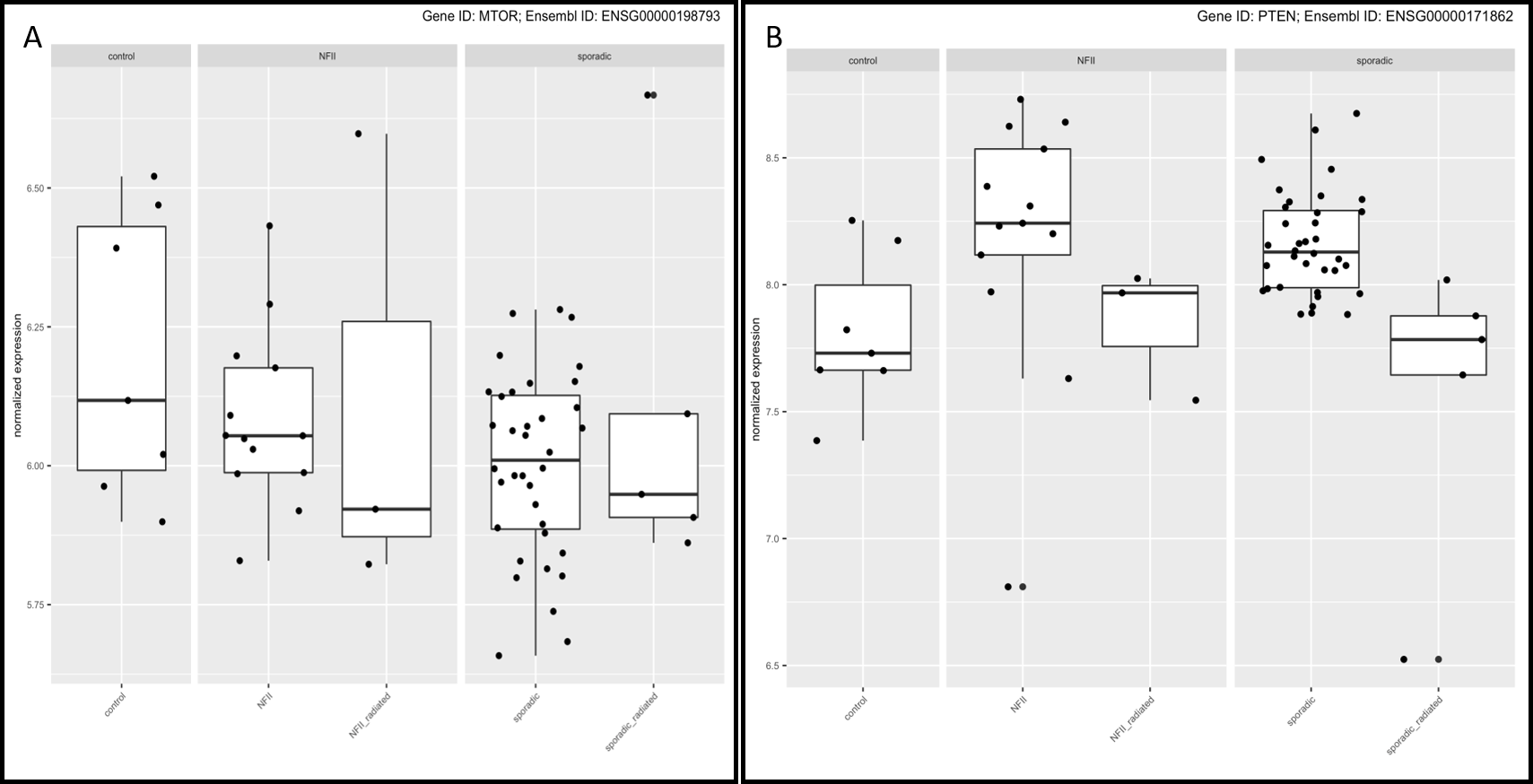


**Figure S1.** Boxplots for *mTOR* (**A**) and *PTEN* (**B**) expression.


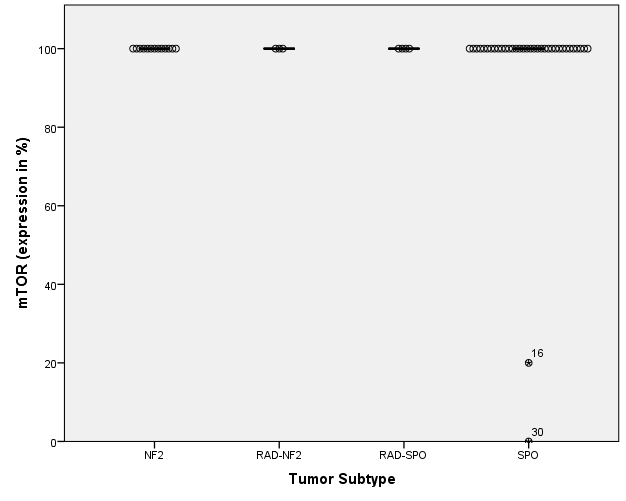


**Figure S2.** Scatterplots of immunohistochemical expression of mTOR.


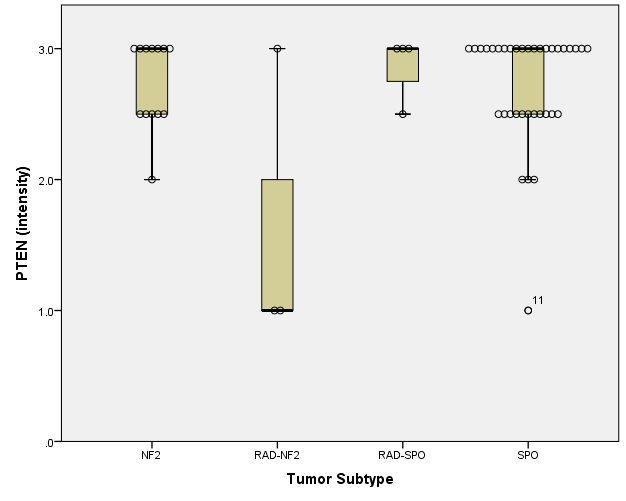


**Figure S3.** Scatterplots of immunohistochemical expression of PTEN.
